# Editorial: Allergens and Allergic Sensitization in Asia and the Tropics

**DOI:** 10.3389/falgy.2021.808044

**Published:** 2021-12-08

**Authors:** Luis Caraballo

**Affiliations:** Institute for Immunological Research, University of Cartagena, Cartagena de Indias, Colombia

**Keywords:** allergen, sensitization, Asia, the tropics, food allergen, house dust mite allergen

The inception and clinical symptoms of allergic diseases are highly dependent on the environment, which makes the existence of a single pathogenesis for all these processes unlikely. Therefore, the search for common or specific components, whether genetic or environmental, will provide important information about basic mechanisms, phenotype characterization, and personalized management; and for this, nothing better than investigating the pathophysiological and epidemiological contrasts of allergies between populations with different genetic backgrounds or between populations with great environmental differences.

The tropical region has been a traditional source of discoveries from foreign and native scientists, and nowadays interesting projects of scientific groups are exploring several aspects of allergic diseases, among them the patterns of sensitization to common allergens and their impact in the disease manifestations, diagnosis, and treatment. These groups take advantage of known aspects of tropical zones such as their natural characteristics of climate and geographical location, their cultural aspects and the fact that most of the countries are not industrialized.

Besides, Asia is a continent with industrialized temperate and tropical countries, as well as important environmental and cultural particularities that could also influence the development of allergic diseases and, of course, these aspects are currently investigated by several groups from this continent, which are collecting valuable data to resolve recognized unmet needs. In fact, while allergic sensitization and the nature of allergenic sources have been extensively characterized in western industrialized countries, such exhaustive analysis is quite lacking and remains to be broadly performed for Asian and tropical countries.

This “Research Topic *Allergens and Allergic Sensitization in Asia and the Tropics*” includes several representative papers on this underexplored field. Here, different peculiarities of allergic problems in Asia and the Tropics are analyzed, which will surely serve as the basis for making comparisons that help to increase knowledge about their pathogenesis.

Three of these works confirm that patterns of sensitization to pollens, indoor allergens, and food in Asia have regional characteristics that make a difference from western countries. For example, in Korea (Jeong and Park), sawtooth oak, and birch pollens in the spring in conjunction with weed pollens of mugwort, ragweed, and Japanese hop are the main causes of seasonal allergic rhinitis. Among food allergens, the sensitization to silkworm pupa and buckwheat is also common in Korean patients. Interestingly, honeybee venom due to apitherapy is an important cause of anaphylaxis. In a study from Thailand (Katel et al.), an Asian tropical country, indoor and outdoor aeroallergen sensitization, as detected by skin test, was observed in 32 and 7.9% of adult allergic rhinitis patients, respectively. Mono-sensitization was found in 16.9% of patients. Mites (65%) and sedge (39.3%) were the most common indoor and outdoor allergens, respectively. Quality of life was also evaluated, showing that allergic rhinitis has a significant impact on QoL of adult Thai patients.

In addition, Wai et al. reviewed the sensitization pattern of seafood (fish and shellfish) allergic diseases in Taiwan, Thailand, Singapore, Vietnam, Hong-Kong, and Japan confirming that these Asian countries have unique ways of food processing and dietary habits that explain the observed differences from western countries. For example, fish and shellfish are eaten raw in some countries that may promote sensitization to heat-labile allergens not otherwise seen in other regions. Fermented fish sauce is commonly used as a condiment in some countries which may promote fish sensitization. Shrimp head and shrimp roe are regarded as delicacies in some Asian countries, but their allergen profiles are yet to be characterized. These three studies show how geographical, social and cultural characteristics influence the types of sensitization and their clinical impact, supporting the need of specific diagnostic tools that allow more accurate and personalized management of allergic diseases (1).

In the tropics, there are also particularities that have been analyzed elsewhere (2). Here, two groups from Ecuador and Colombia studied several aspects concerning the emergence of allergic sensitization in children and the emergency room visits of wheezers living in tropical environments respectively. Cooper et al. analyzing a large Ecuadorian birth cohort found that skin prick test positivity starts with mite and is followed by cockroach. The risk of mite sensitization increased with maternal sensitization, while household overcrowding at birth, rural residence, birth order, intestinal helminth decreased this risk. In contrast, Zakzuk et al. had found that indicators of unhygienic conditions were risk factors for house dust mite and Ascaris sensitization in children, using both extracts (3) and recombinant allergens (4). The reason of these contrasting findings deserve a more detailed analysis of the rural vs. urban children populations. Also, Muñoz et al. in this Research Topic present finding indicating that the risk of emergency room visits and intensive care unit admissions for wheezers 2–6 years old increased with poverty indicators such as lack of tap water and sewage, as well as cohabiting with two or more siblings. The origin of these associations was not evaluated but probably depend on respiratory viral infections, which supports the proposal of a more comprehensive and personalized evaluation of wheezing children (5).

Regarding the diagnosis of allergy in the tropics, in their paper, Mourao et al. demonstrate that allergen conjunctival provocation test is safe and reproducible if standardized allergens are available. They use a conjunctival provocation test to define the clinical impact of sensitization to *B. tropicalis* in patients with allergic rhinoconjunctivitis; which is an advance in defining the allergenic activity of allergens and is an important step for obtaining an appropriate diagnostic tools for precision allergology in the tropics (6).

The Research Topic also includes the necessary theme of component resolved diagnosis (CRD) reagents for regional diagnosis of allergy sensitization, and this was investigated by Wangorsch et al. using recombinant *P. americana* allergens and showing that the most frequent inducer of sensitization in a tropical country like Venezuela was Per a 7, a place where sensitization to Per a 2, Per a 5, and Per a 10 was not found. In addition, being mosquito allergy important in the tropics, Cantillo and Puerta discusses the current knowledge about mosquito allergy, allergens, cross-reactivity, and proposals of component resolved approaches based on mixtures of purified recombinant allergens to replace saliva-based or whole-body extracts.

Another important allergen that exhibits cross-reactivity among similar molecules from house dust mite and intestinal helminths such as *Ascaris lumbricoides* and needs to be included in regional platforms for CRD, is glutathione transferase (GST), which is reviewed by Zakzuk et al. GST allergens belong to different classes: mu (Blo t 8, Der p 8, Der f 8, and Tyr p 8), sigma (Bla g 5 and Asc s 13), or delta (Per a 5). In this review, some aspects of the biology of GST, mainly their allergenic activity, structural aspects and the clinical impact of their cross-reactivity are analyzed.

In conclusion, the scientific works of this Research Topic cover different aspects of the pathogenesis, mainly the allergen sensitization, in two regions with geographic, cultural, socioeconomic and genetic particularities; showing interesting results that will help to better understand allergic diseases and improve precision allergology in Asia and the Tropics. Although not the focus of this Research Topic, it is important to keep in mind that the sensitization process is being affected by the climate change (7); therefore we have to be aware of potential changes on the epidemiological trends of allergic diseases.

## Author Contributions

LC conceived and wrote the manuscript.

## Funding

This study was supported by Grant 803-2018 (Minciencias, Colombia).

## Conflict of Interest

The author declares that the research was conducted in the absence of any commercial or financial relationships that could be construed as a potential conflict of interest.

## Publisher's Note

All claims expressed in this article are solely those of the authors and do not necessarily represent those of their affiliated organizations, or those of the publisher, the editors and the reviewers. Any product that may be evaluated in this article, or claim that may be made by its manufacturer, is not guaranteed or endorsed by the publisher.
